# Mathematization of nature: how it is done

**DOI:** 10.1007/s00422-021-00914-5

**Published:** 2021-11-27

**Authors:** J. Leo van Hemmen

**Affiliations:** Physik Department T35, Technische Universität München, 85747 Garching bei München, Germany

**Keywords:** Mathematization, Core concepts, Scales, Scaling hypothesis

## Abstract

Natural phenomena can be quantitatively described by means of mathematics, which is actually the only way of doing so. Physics is a convincing example of the mathematization of nature. This paper gives an answer to the question of how mathematization of nature is done and illustrates the answer. Here nature is to be taken in a wide sense, being a substantial object of study in, among others, large domains of biology, such as epidemiology and neurobiology, chemistry, and physics, the most outspoken example. It is argued that mathematization of natural phenomena needs appropriate core concepts that are intimately connected with the phenomena one wants to describe and explain mathematically. Second, there is a scale on and not beyond which a specific description holds. Different scales allow for different conceptual and mathematical descriptions. This is the scaling hypothesis, which has meanwhile been confirmed on many occasions. Furthermore, a mathematical description can, as in physics, but need not be universally valid, as in biology. Finally, the history of science shows that only an intensive gauging of theory, i.e., mathematical description, by experiment leads to progress. That is, appropriate core concepts and appropriate scales are a necessary condition for mathematizing nature, and so is its verification by experiment.

## Introduction: What is the question?

The key question whose answer we want to analyze in this short essay is how the mathematization of nature works. That it works is clear, as many concrete, even famous examples show, but how is it done? In answering this question, we will restrict ourselves to the natural sciences.

Mathematization of nature means that we can mathematically describe natural phenomena at hand and quantify the processes we analyze. In short, numbers in, numbers out. Only mathematics allows us to do so. Since four centuries, physics has shown that mathematization works in the sense that it is highly successful. Far more important, however, is the fact that without its mathematization the impact of physics would have been minor because making quantitative predictions that can be verified experimentally not only allows an internal check that speeds up progress in understanding (through feedback) but also gives rise to applications that can be precisely tailored to the situation at hand. Dijksterhuis (1961) brilliantly described the significant contributions of Johannes Kepler, Galileo Galilei, and Simon Stevin to the mechanization of our (physical) world picture that reached its final foundation, its crowning achievement, in the work of Isaac Newton (1687).

For Dijksterhuis (1961), mechanization meant the development of physics in general, since until the seventeenth-century mechanics was its dominant part. Hence, his focus was on mechanics. His classic (Dijksterhuis 1961) shows that mathematization as mathematical description of physical reality concretized as late as about 1600 with Galileo and Stevin, though the first ideas about mechanics already appeared two millennia earlier.

We will take advantage of some concrete but simple examples to illustrate the essential role that core concepts play in reaching such a mathematical description of natural reality. (In the present context, core concepts were also called key concepts but from now on the expression core concepts will be used. A key opens a door and a core is the central part carrying the full weight.) After having consolidated our common understanding, we will turn to verifying how core concepts in conjunction with suitable scales play their essential role in the success of a few but much acclaimed examples taken from *Biological Cybernetics*, the world’s oldest journal in computational neuroscience. The journal dates back to 1961/63. For instance, Horace Barlow (Cambridge University) and Norbert Wiener (MIT; Cambridge, MA) belong to its founding fathers (van Hemmen 2009). A short outlook will gather the insights we have gained.

Science is a quest, a reconnoitering expedition to find so-called logical explanations of phenomena occurring in the world around us. Such a quest is akin to looking for points of orientation and then tracing the outline of an as-yet-unknown landscape. It should be constantly borne in mind that many erudite and learned arguments fill the pages of books on the history and philosophy of science but that here we will simply skip these and pick a few masterpieces or introductions as sign posts in a fascinating landscape. As for the history of mechanics as it leads through many loopholes to Newton’s laws (1687), the classic *The mechanization of the world picture* of Dijksterhuis (1961) will provide the reader with practically all the details needed, and many more. From a more general perspective, the monograph of Simonyi (2012) paves the way to a grandiose overview of more recent times. For the present purposes, Okasha’s booklet (Okasha 2002) suffices as a nice, succinct, philosophical-background reference, also mentioning useful supplementary literature.

## The solution: core concepts and scaling hypothesis

It is time to delve into the rich soil of concrete examples that illustrate the relevance of core concepts in the context of the scaling hypothesis (van Hemmen 2014). We discuss a few core concepts as they play their key role. First, we turn to Newton’s second law because nearly everyone knows this example and can now recognize it as a paradigm. Next, we quickly analyze three examples taken from theoretical neuroscience, viz. a neuron as threshold element, STDP as a canonical learning paradigm, and the population vector code as determinant of motion. All four examples are only valid on a certain scale, which naturally leads us to the scaling hypothesis.

### Core concepts

We embark on analyzing four core concepts. We will then discover that there is a natural scale beyond which our natural laws do not hold.

*Momentum & Newton’s laws* Many people may remember the cannonball problem from their days in high-school or grammar school: A cannon is placed on a tower of height *h* and, at time $t=0$, a cannon shoots a cannon ball of mass m and velocity vector $\mathbf {v} = (v_1, v_2, v_3)$, pointing upward in some direction. Now solve the problem of determining the ball’s orbit after leaving the cannon and neglecting friction. As we live in a three-dimensional world, the velocity $\mathbf {v}$ and a direction have three components; though accidental, for the present problem two will do as the ball moves in a two-dimensional, vertical, plane spanned by the tower and the direction vector. The reader may remember that, neglecting friction, a parabola was the orbit one was looking for and that this result followed from Newton’s second law. How did that work?

Newton (1687) formulated his three laws in a monumental work with far-reaching consequences, also for planetary motion. For background information, see the classical work of Dijksterhuis (1961). Newton’s second law—commonly known as “force equals mass times acceleration”—describes how a particle with mass *m* and velocity $\mathbf {v}$ moves in three dimensions under the influence of a force $\mathbf {F}$. Three ingredients are to be noted. First, the force $\mathbf {F}$ is, like $\mathbf {v} = (v_1, v_2, v_3)$, a vector $\mathbf {F} = (F_1, F_2, F_3)$ with three components since the space in which we live has three dimensions.

Now we need a totally new concept, a core concept, viz. the momentum $\mathbf {p} = m \mathbf {v}$, which took physics two millennia to discover (Dijksterhuis 1961). It was Simon Stevin (1548–1620) who discovered—almost a century before Newton—the importance of momentum (Dijksterhuis 1970) in his collision experiments on a frictionless table: The total momentum is conserved. That means that for two (round) disks with momentum $\mathbf {p}$ the sum $\mathbf {p}_1 + \mathbf {p}_2$ is the same before and after the collision; i.e., it is conserved.

Imagine you were an unprejudiced observer around 1600. Along comes Stevin who joyfully tells you that total momentum is conserved in collision experiments. You would turn up your nose and ask yourself: What does this nonsense mean? Mass *m* has dimension kg and I now need to multiply *m* by a weird vector $\mathbf {v}$ of dimension m/s in order to get the momentum $\mathbf {p} = m \mathbf {v}$. Then, along comes Newton (1687) who brings in a force $\mathbf {F}$ and gives the whole thing a meaning by posing $\mathbf {F} = \mathrm {d}\mathbf {p}/\mathrm {d}t$, Newton’s second law, where (à la Leibniz) d/d*t* means differentiation with respect to the time *t*, a new mathematical idea he brought up independently of Leibniz.

$\mathbf {F} = \mathrm {d}\mathbf {p}/\mathrm {d}t$ is what most people know but need not be aware of yet because for a particle with mass *m*, position vector $\mathbf {x} = \mathbf {x}(t)$, where $\mathbf {x}$ generally depends on the time *t*, and velocity vector $\mathbf {v} = \mathrm {d}\mathbf {x}/\mathrm {d}t$, we get $\mathbf {F} = \mathrm {d}\mathbf {p}/\mathrm {d}t = m \mathbf {v}/\mathrm {d}t \equiv m \mathbf {a}$, mass times acceleration $\mathbf {a} = \mathrm {d}^2\mathbf {x}/\mathrm {d}t^2$ with $\mathbf {p} = m \mathbf {v}$ and the mass *m* constant. Newton’s second law is a universally applicable law of nature. That is, there is no exception...as long as $v/c \ll 1$ where $v = \Vert \mathbf {v}\Vert$ is the particle’s velocity (in m/s) and *c* is the velocity of light. Already here is the scale disguised as relativity theory lurking in the background.

It is worth noting that, simply put, $\mathbf {F} = \mathrm {d}\mathbf {p}/\mathrm {d}t$ describes the change in momentum $\mathbf {p}$ under the influence of a force $\mathbf {F}$ over time. Mathematics allows us to solve the differential equation $\mathbf {F} = \mathrm {d}\mathbf {p}/\mathrm {d}t$; rarely explicitly, but always numerically. The equation $\mathbf {F} = \mathrm {d}\mathbf {p}/\mathrm {d}t$ immediately gives the solution to the cannonball problem as $\mathbf {F} = (0, 0, -g)$ where $g = 9.81$ m/s$^2$ is the gravity constant and $\mathbf {x}(0) = (0,0, h)$ because the cannon was standing on a tower of height *h*. Newton’s second law also explains the conservation of momentum as Newton postulated his third law as well: $\textit{actio} = -\textit{reactio}$ during collisions. The sum $\mathbf {F}_\mathrm {total}$ of all forces on and, for the collision, in the plane of the (frictionless) table therefore $= 0$ so that $\mathrm {d}(\mathbf {p}_1+\mathbf {p}_2)/\mathrm {d}t = \mathbf {F}_\mathrm {total} = 0$ and $\mathbf {p}_1+\mathbf {p}_2$ is conserved. That is, the conservation of momentum follows.

Neuron as threshold element It is time to turn to biology. More in particular, to computational neuroscience. Let us first focus on one of the, also historically, first core concepts, the neuron as threshold element. At the axon hillock, the axon “leaves” the neuron so as to deliver a neuron’s output to elsewhere. Dropping nearly all historical and other details (Ermentrout and Terman 2010; Koch 1999), we simply state as an experimental fact that once the membrane potential *V*(*t*) exceeds a certain threshold $\theta$, i.e., as soon as $V(t) > \theta$, the neuron produces a spike, a huge—as compared to the usual mV fluctuations—positive potential jump of about 0.1 V amplitude as compared to the resting potential and lasting for about 1 ms.

Action potentials, or spikes for short, are generated through the coordinated activity of many ion channels (Ermentrout and Terman 2010; Koch 1999). There is little doubt that single ion channels can be described in surprising detail in the context of biological physics. However, a concerted action of hundreds of ion channels generating a spike is still beyond the horizon of theoretical neurobiology and theoretical biophysics. Accordingly, our scale (sic) is here the neuronal and not the ion-channel one, and we focus on the neuron as a threshold element, meaning that it produces an action potential once its membrane potential *V* exceeds a certain threshold $\theta$, the core concept.

A neuron was treated as abstract notion of threshold element as early as McCulloch and Pitts (1943). McCulloch–Pitts neurons operate in discretized time with 1 ms time bins and outputting either a 1 for an active state, meaning spike emission, or 0 for an inactive state. (The McCulloch–Pitts paper has been quoted by many but read by hardly anybody as the arguments are embedded in the heavily formal language of logic.)

The threshold behavior in conjunction with the spike, which they called the “overshoot,” has also motivated Hodgkin and Huxley to perform their now famous experiments (Hodgkin and Huxley 1952; Huxley 2002; Meunier and Segev 2002). Their work, which was both experimental and theoretical, earned them the Nobel Prize and initiated an overwhelming plethora (Ermentrout and Terman 2010; Koch 1999) of highly detailed neuron models describing many different situations, all outputting different spike shapes, but—and that is the cardinal issue—exhibiting *threshold* behavior for the potential *V*. Their system of four coupled nonlinear differential equations that contains the threshold only implicitly is the result of their brilliant fitting work, dating back to the early fifties, when computers were still *in statu nascendi*. This is an intellectual achievement that theoreticians can hardly overestimate.

Spike-timing-dependent plasticity (STDP) and its learning window The barn owl (Konishi 1993) is a nocturnal predator that azimuthally localizes its prey in the woods with a precision of $1^{\circ }{-}2^{\circ }$. Azimuthal sound localization uses the time difference between left and right eardrum as direction coding, which therefore depends on the interaural distance *L* between the two; at present, $L \approx 6$ cm. This time difference is washed out strongly by a wide distribution of delay times before the spikes stemming from left and right eardrum finally meet the neurons at the laminar nucleus, where the first map is to be built through synaptic learning, unless genetic coding would reach such a spatial $\upmu$s precision, which is beyond scientific imagination, to phrase it friendly.

The usual precision one expects from a bird of which the neuronal system operates with spikes is in the millisecond range but a simple calculation shows that in azimuthal sound localization a barn owl reaches a precision in the $\upmu$s range, three orders of magnitude better than the ms $=$ millisecond one expects. This is the Konishi paradox. To solve it, spike-timing-dependent plasticity (STDP) was invented (Gerstner et al. 1996). Its experimental verification (Markram et al. 1997) appeared more than a year later and illustrates that theory may well precede its experimental confirmation. It is far more important, however, that a theory, mathematical (or not), *does* allow experimental verification, which need not always be the case (Smolin 2006).

**Fig. 1:**
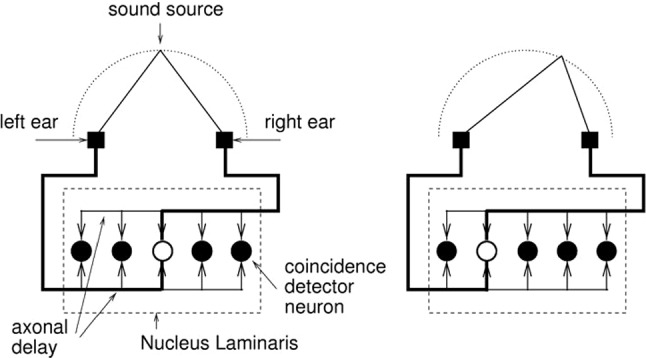
Jeffress model. Schematic anatomy of the barn owl’s laminar nucleus, the first station where axons from left and right ear come together. The figure shows the Jeffress (1948) idea of converting a time code into a place code: Depending on the direction, different neurons as threshold-coincidence detectors simultaneously get input from left and right so that different directions “ignite” different neurons. The latter are positioned in a one-dimensional row, as are the directions. In reality (Carr and Konishi 1990) there are many more (i.e., about 20) neurons than the 5 shown here and many more axons, all parallel to each other, coming from left and right and accidentally connecting each other though synapses that are “in between,” as indicated above

The STDP basics is simple to explain (Kempter et al. 1999; van Hemmen 2001) and goes as follows. Experimentally, it is known that the young barn owl, once it is able to leave the nest three weeks after hatching, cannot perform the azimuthal sound localization to a precision of $1^{\circ }{-}2^{\circ }$ it would need to survive. After two more weeks, however, it can. What happens during these two weeks?

Synapses need time to develop, to “learn,” which in the barn owl happens during these two critical weeks. In the barn owl’s laminar nucleus, axons stemming ultimately from left or right cochlea and carrying the interaural time-difference (ITD) code through a certain time delay meet for the first time. Let the interaural or, more precisely, the inter-tympanic distance be *L* and the direction of the sound source with respect to straight on be $\theta$ so that straight on means $\theta = 0$, then the ITD equals $(L/v_s) \sin \theta$ where $v_s$ is the velocity of sound, about 330 m/s. The time code of the prey (or predator in case of the barn owl’s meal) direction is then contained in $L \sin \theta$. No more, no less. And the brain has to decode this and tell the animal what to do. In other words, the brain apparently converts the time code contained in $\theta$ into a place code by letting neurons fire when they get their maximal input, i.e., simultaneously from left and right ear. (As the cochlea is in between, this always means modulo the period *T* of the oscillation.) This is what the anatomy is going to do. The original, so to speak theoretical, idea of converting time code to place code is due to Jeffress (1948), who published it long before its anatomical confirmation appeared (Carr and Konishi 1990).

Returning to the anatomy that is depicted schematically in Fig. 1, the (vertical, fast) axons turn left or right, so to speak, so as to run parallel to each other with the spike speed now being slowed down and contacting about 20 neurons in a row through excitatory synapses. One needs to keep in mind that here there is no inhibition. The whole anatomical construction is genetically predisposed, though only on a global level, but the barn owl’s $\mu$s precision is not. By construction, the growth of this anatomical construction depends among others on available food, which fluctuates from day to day, and growth of thousands and thousands of axons connected to the cochlear frequency decomposition, so that genetic coding is out. Now STDP with its learning window *W* as core concept comes in.

**Fig. 2:**
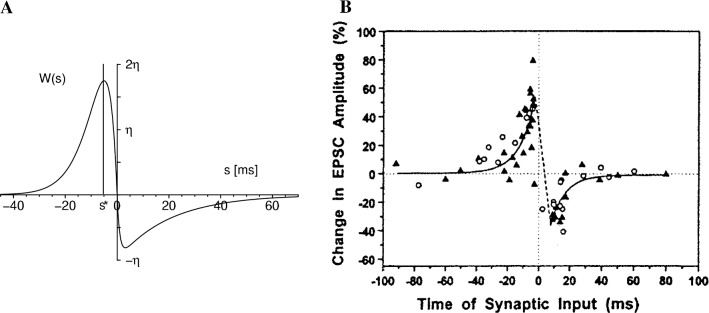
A. STDP’s learning window *W* in units of the learning parameter $\eta$ as function of the delay $s=t^f_i-t^n$ between presynaptic spike arrival at synapse *i* at time $t^f_i$ and postsynaptic firing at time $t^n$. If *W*(*s*) is positive (negative), for some *s* the synaptic efficacy $J_i$ is increased (decreased). The increase of the excitatory $J_i$ is most efficient, if a presynaptic spike arrives a few milliseconds *before* the postsynaptic neuron starts firing (vertical line at $s=s^*$). For $|s|\rightarrow \infty$ we have $W(s)\rightarrow 0$. Taken from Kempter et al. (1999). B. Experimentally obtained learning window of a cell in rat hippocampus; reprinted by permission (Zhang et al. 1998). The similarity to the left figure is evident. It is important to realize that the width of the learning window is to be in agreement with other neuronal time constants. In the auditory system, for instance, these are nearly two orders of magnitude smaller than, e.g., in hippocampus so that the learning window’s width scales accordingly

We focus on synapse *i* with efficacy $J_i$ and positioned on a certain neuron in the row shown in Fig. 1 and specify how it will change depending on the arrival time $t_i^f$ of a spike at the synapse and the firing time $t^n$ of the postsynaptic neuron it is on. This specification, namely, is the learning window *W*, a function that can assume either positive or negative values. It is the key to understanding the benign influence of *W* on the formation of the barn owl’s extremely precise azimuthal map. The speed of this kind of learning can be tuned mathematically by a prefactor $\eta$ so that the actual change is described by $\Delta J_i = \eta W$. The learning window $W = W(s)$ with $s = t_{i}^{f}-t^{n}$ has a shape as specified by Fig. 2. For now its precise shape is not important. Only its qualitative appearance counts. That is, $W(s) \ge 0$ is positive for $s = (t_{i}^{f}-t^{n})< 0 \Leftrightarrow t_{i}^{f} < t^{n}$ so that the presynaptic spike arrives *before* the postsynaptic neuron fires. On the other hand, $W(s) \le 0$ is negative for $s = (t_{i}^{f}-t^{n}) > 0 \Leftrightarrow t^{n} < t_{i}^{f}$ so that the presynaptic spike arrives *after* the postsynaptic neuron has fired. Colloquially, those who come too late shall be punished.

**Fig. 3:**
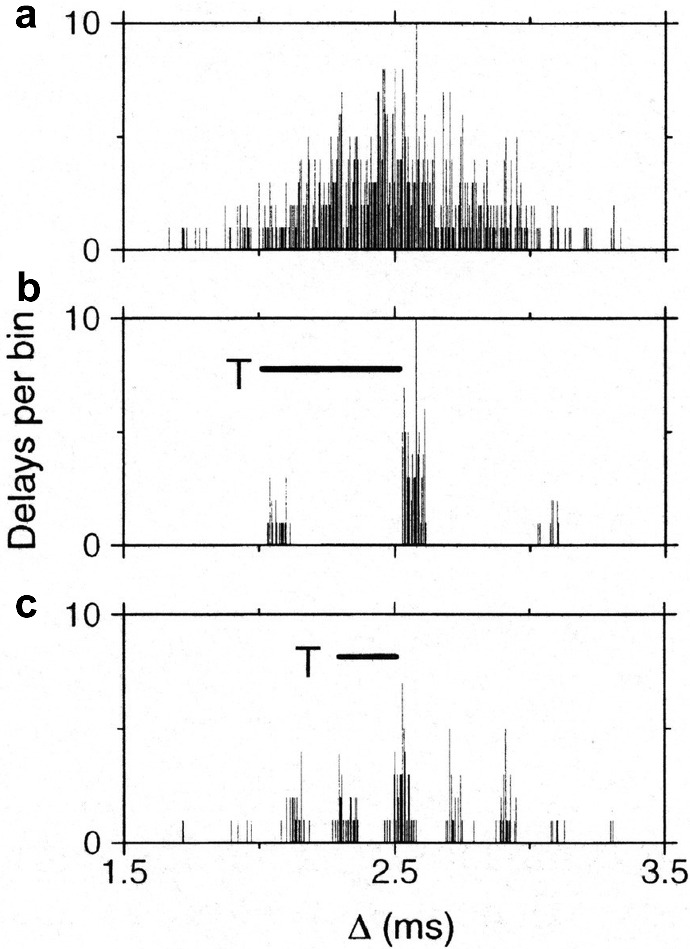
Evolution of synaptic strengths and “survival of the fittest” in the young barn owl’s laminar nucleus during its critical period. **a** Before learning, there are 600 synapses with a Gaussian distribution ($2.5 \pm 0.3$ ms) of the synaptic strengths connected to a single neuron, binned as a function of the signal-transmission delays w.r.t. arrival at left and right eardrum. **b** After a learning session from a stimulus of 2 kHz, only 105 synapses survive. They have delays that differ by multiples of the period $T = 500\,\upmu \hbox {s}$ (scale bar); phrased differently, modulo the period *T* everything in an oscillation is identical. The output spikes exhibit phase locking with a vector strength (van Hemmen 2013) of 0.97 corresponding to a temporal precision of $20\,\upmu \hbox {s}$. **c** Analogous to **b** but now for an input frequency of 5 kHz. Figure and data taken from Gerstner et al. (1996), Fig. 2, who also show how in subsequent nuclei the temporal precision can be increased by an order of magnitude

During map formation, many synapses on a neuron in the laminar nucleus are steered by coherent input of a specific frequency stemming from the frequency decomposition performed by the cochlea of left *and* right ear(drum)s. For a specific rotation angle $\theta$ of the head, the time delay between left and right eardrum is fixed but at the start of the critical period of two weeks for synaptic learning in the barn owl’s brain there is a huge scatter in time delays along different axons. In general, this wetware cannot change but the synapses can. Figure 3 shows what happens as time proceeds: The “good” ones among the synapses are strengthened and the “bad” one are weakened, which is due to the positive *and negative* part, respectively, of the learning window *W*.

Because in the barn owl phase locking of the cochlear neurons happens up to 9 kHz, the temporal resolution is far better than in, say, human ears. (Though phase locking in humans and most other vertebrates is restricted to $\le 1.5$ kHz, humans have more brains, through which they compensate their wetware deficit and become as good as barn owls, with a spatial resolution of $1^{\circ }{-}2^{\circ }$ for azimuthal sound localization.) Though in the case of the barn owl’s laminar nucleus all synapses are excitatory, in the auditory system of mammals inhibition plays a role as well and STDP can be adapted accordingly; see, e.g., Leibold and van Hemmen (2005).

Not only does STDP describe the learning dynamics of individual synapses in that it tunes them so as to fit their spatiotemporal surroundings but, as Fig. 3 shows for a single neuron, it also governs how many synapses and which ones operate in concert so as to build a topographic map. To obtain a full map, however, synapses on different neurons also need—so to speak—to tell each other what they are doing, through a kind of retrograde signaling (Fitzsimonds and Poo 1998). In the barn-owl case (Kempter et al. 2001), this means that along the string of parallel axons coming from left and right ear the synapses connected to the very same presynaptic axon communicate their positive or negative change to their neighbors and in this way influence each other, a mechanism called axon-mediated synaptic leaning (AMSL). That is, given a suitable anatomy an ITD map with the required topographic precision emerges from a combined action of homosynaptic spike-based Hebbian learning through STDP together with AMSL as its propagator along the presynaptic axons.

As a final note, STDP also gives rise to (Wenisch et al. 2005) a full explanation of how direction-selective spatiotemporal maps come about in primary visual cortex V1. The interplay of the anatomy of excitation and slightly longer-ranged inhibition and STDP is essential to giving rise to a spatiotemporal map. This fact shows again the potency of STDP as core concept in synaptic learning and map formation (van Hemmen 2006). As shown by Wenisch et al. (2005), map formation in V1 is mainly a matter of self-organization based on specific neuroanatomy in conjunction with STDP that then gives rise to the spatiotemporal receptive fields from which the whole map arises, as confirmed by experiment.

Population vector code The population vector code relates directional tuning of single cells to global, directional motion induced by an assembly (Hebb 1949) of neurons. The scale we now focus on is that of an assembly of neurons; in this case, in motor cortex (Georgopoulos et al. 1986; van Hemmen and Schwartz 2008). The underlying geometric idea is appealingly simple and its predictions are extremely powerful. Let us assign to each motor neuron with label *i* its preferred direction $\mathbf {e}_i$, a unit vector. It is a priori not evident that one can assign such a vector but experimental evidence has shown one can (Georgopoulos et al. 1986; van Hemmen and Schwartz 2008). For an assembly (Hebb 1949) or population of motor neurons $\{1 \le i \le N\}$ with momentary firing rate $\nu _i = \nu _i(t)$, the weighted vector sum, the so-called population vector $\mathbf {n}$

1 $$\begin{aligned} \mathbf {n} \equiv \nu \mathbf {e} = \sum _{i = 1}^N \nu _i \mathbf {e}_i , \end{aligned}$$

encodes the direction $\mathbf {e}$ of movement resulting from an assembly of motor neurons, while $\nu$, the length of the population vector $\mathbf {n}$, is proportional to the instantaneous speed of the drawing motion we focus on.

That is, $\mathbf {n}$ predicts the grasping direction or the direction an animal would like to choose (van Hemmen and Schwartz 2008) for catching its prey, be it the barn owl (*Tyto alba*), the back swimmer (*Notonecta undulata*) or the sand scorpion (*Paruroctonus mesaensis*). In fact, for all these animals it has been shown that the population vector applied to the sensory instead of the motor system of the animal already predicts its prey-catching behavior (Stürzl et al. 2000; van Hemmen and Schwartz 2008; van Hemmen 2014). In other words, the population vector code functions as a neuronal actuator. An extra advantage is that sensory and motor system would use the same coding, which makes this *correspondence principle* even more plausible.

### Scaling hypothesis

The scaling hypothesis (van Hemmen 2014) formulates what is known from many examples, but since a definitive proof cannot be provided we are forced to formulate it as hypothesis: There is a scale on and not beyond which a specific description holds. Different scales need different conceptual and mathematical descriptions. We have just seen a few examples as illustration in that the previous core concepts only function on a certain scale. The population vector code functions on the population level is three orders of magnitude larger than the neuronal one of threshold principle and STDP.

The most outspoken examples of the correctness of the scaling hypothesis are still provided by physics. It is meanwhile known that Newton’s second law $\mathbf {F} = \mathrm {d}\mathbf {p}/\mathrm {d}t$ only holds for (i) velocities $|\mathbf {v}|/c \ll 1$ and (ii) spatial scales down to $0.1\,\upmu \hbox {m}$. Six orders of magnitude smaller than our macroscopic world (of 1 m), i.e., in the nm range, dynamics is governed by quantum mechanics, which has some relations to our macroscopic world but also needs new and totally different principles such as its probability interpretation that have no resemblance to what we are macroscopically used to. That is, these principles cannot be derived but must be taken as “naturally” given, as is Newton’s law. Adding relativity one obtains an even richer structure. Again six orders of magnitude smaller, we arrive at quantum chromodynamics (QCD) and the so-called standard model, viz., of elementary articles and quarks, their mathematical description stemming from quantum field theory. What is beyond, be it space or time (think of origin of the universe) is a heavily debated domain of research.

### Core concepts are—usually—only valid on a certain scale and stand by themselves

It is time for a tentative summary. We have seen that core concepts are a necessary condition for the mathematization of nature. They need to be “rightly” chosen, which may take time; even lots of time (Dijksterhuis 1961). Furthermore, their validity is bound to a certain scale in space and/or time, beyond which they do not hold. It may also take lots of patience (Simonyi 2012) to discover such a scale. One really outspoken example may do for now.

More than a century ago physicists discovered in the context of radiation—think of Max Planck’s 1900 introduction of his novel constant $h = 2\pi \, \hbar$—that Newton’s mechanics apparently does not hold on the scale of atoms and molecules. The question of what could replace classical mechanics tantalized physics for three decades before a novel kind of physics, since then called quantum mechanics, was discovered during the years 1927–1929. The corresponding probabilistic interpretation, most notably, the highly successful one due to the Kopenhagen School of Niels Bohr, has been confirmed experimentally in all details, but nevertheless even the genius of Einstein was not able to accept it (mainly because of his misinterpretation of the notion of chance). As noted, beyond quantum mechanics, on a scale six orders of magnitude smaller, we enter the QCD domain. Each domain has its own rules, which cannot be derived from the “coarser” one, despite having relations with it. That is, the rules exist in their own right.

Neuroscience also has many scales, viz. that of ion channels, that of neurons, that of assemblies of neurons,.... Their scales are separated by orders of magnitude. There are relations, maybe even intimate ones, between the descriptions on different scales. Here we do not aim at ‘scales’ in the technical sense of nonlinear dynamics but at those as they exist for instance between classical mechanics à la Newton and quantum mechanics; see, e.g., van Hemmen (2014). These different scales have descriptions *in their own right*. They cannot be derived fully from theories that are valid on a larger or smaller scale, be that in space or time. This is what one may call the ‘principle of scientific independence’ or, for short, the independence principle.

For example, the probabilistic interpretation of quantum mechanics exists in its own right. Einstein may have grumbled “God does not play dice” but the simple reply is: Why not? Deciding that is neither up to Einstein, nor to you, nor to anybody else. Only experiment decides. End of the discussion.

From the present point of view, (many of) the “laws” of psychology exist in their own right and there is little hope that they will ever be straightforwardly “derived” from neuroscience, which focuses its attention on much smaller scales. That is, there are doubtlessly many relations between phenomena on the neuronal and, hence, also on the macromolecular level—c.f. neuromicrobiology—and the behavior of humans, and other animals, but psychology has several independent(ly existing) notions describing behavior that only exist by themselves—on the macroscopic scale of psychology. Freud sends greetings.

## More core concepts from theoretical neurobiology

It is now time to harvest core concepts while noticing the appropriate scales. We do so by analyzing some highly acclaimed papers that have appeared as ripe, tasty, fruits in *Biological Cybernetics*, the world’s oldest journal in computational neuroscience, which has meanwhile reached the respectable age of 60 years and 115 volumes. Nearly all of these examples exhibit a core concept and do so in the context of a specific scale. For additional comments, see Koenderink (2021), Kelso (2021), Wilson and Cowan (2021), Baccalá and Sameshima (2021), Humphries and Gurney (2021), von der Malsburg (2021) and Abarbanel (2021).

Scale space Eyes behold the three-dimensional world through a two-dimensional projection onto the retina, an image. Images may be blurred or distorted, for instance, because of unlucky illumination of a scene, or bad quality of the eye lens, or noise, or a combination of all together. Since the mathematical theory of image processing is of fundamental importance—not only to vision!—Koenderink (1984) apparently chose Biological Cybernetics (BC) for publishing his essay “The structure of images.”

Here he implemented—cf. Koenderink (2021)—the core concept of *scale space* that Witkin (1983) had introduced the year before. In doing so, he noticed how important Gaussian were in this game and realized that Gaussians have a unique property that comes in a minute. Introducing the mathematical framework of the diffusion equation for functions defined on the plane $\mathbb {R}^2$, he developed a full-blown theory incorporating and explaining, and in this way integrating, many experimental facts that were already known. His bright observation was that a Gaussian is the Green’s function of a two-(or *n*)-dimensional Laplacian, so to speak the infinitesimal generator of blurring. [A Green’s function or fundamental solution (Evans 2015, §2.3, Eq. (13)) is an integral kernel $\Phi$ providing an explicit solution to, e.g., $(\partial _t - \Delta ) u = f$ with initial condition $u(t=0)=0$ through $u (\mathbf {x}, t) = \int \mathrm {d}\mathbf {y} \mathrm {d}s \, \Phi (\mathbf {x} - \mathbf {y}, t -s) f(\mathbf {y},s)$.]

In summary, here the core concept is ‘scale space’ and the scale of image analysis is macroscopic, $\mathbb {R}^2$ or, if you like, the retina plane.

Haken, Kelso, Bunz & the HKB model The best you can do in getting famous or at least well known in science is inventing a model that carries your name. Good papers in BC need a while to take off, meaning that their content is novel, but they then fly quite long, which in fact is a proven characteristic of many BC papers.

Haken et al. (1985) invented a model that now carries their names, viz., HKB. They did not derive but simply posed it so as to mathematically describe hand movements. It is a mathematically simple looking model of two coupled nonlinear ordinary differential equations of the oscillator type, with a bit of noise to incorporate many unknown, external, influences, and describing hand movements; it can be reduced to a single equation for the relative phase between two fingers. The HKB equations contain some cleverly chosen parameters that allow reproducing some experiments that Kelso and coworkers had performed and published a few years earlier. Many more experiments followed, a few of which are described and commented by Kelso (2021).

Clearly, the scale is macroscopic and so is that of the equations. The question of how to derive equations of the HKB type neuronally is still a challenge but, as argued in Sect. 2.3, this may, but need not, happen. What then remains is a valuable quantitative description on a certain scale, the macroscopic scale of hand movements.

Wilson–Cowan equations and the continuum limit Neurons are discrete entities in a three-dimensional continuum. On the other hand, continuum descriptions allow application of powerful mathematical techniques related to the mathematics of pattern formation (Hoyle 2006). The Wilson and Cowan (1973) equations offer a continuum description of neuronal reality consisting of continuum variables that describe two populations of excitatory and inhibitory neurons. As such they are meanwhile well-known and widely used. As Wilson and Cowan (2021) confessed, they aimed from the very beginning to, among others, traveling waves as possible solutions—in view of nature, rightly so. The scale is, say, neuronal and so are the equations.

Hallucinations, a popular topic in the seventies, provide a nice example and the Wilson–Cowan equations were shown (Wilson and Cowan 2021) to allow the typical hallucinatory patterns as solutions. One need to admit, though, that the continuity of space is immaterial as a lattice of discrete neurons allows the very same patterns as solutions (Fohlmeister et al. 1995).

Neurons being discrete entities, a far more fundamental question is whether the popular Wilson–Cowan equations could be mathematically derived from a discrete model through a continuum limit. There are examples in applied mathematics, as *tour de force* far beyond the present context, that allow such a limit. Nevertheless, an early ansatz in this direction of a rigorous proof already exists since long (van Hemmen 2004).

Partial directed coherence = PDC Uncovering coherence such as the simultaneous, i.e., within a small time window, spiking of many neurons has been proven invaluable to neuroscience for understanding collective behavior under the influence of homogeneous (e.g., a pure tone) or correlated input. One does so by analyzing many-neuron time series of very many spikes that are considered as point events. Machine learning and, thus, high computer power are meanwhile essential in sorting the huge amounts of spike data.

Because of the inherent uncertainty that experimental data contain, statistics comes in as well. And one needs sorting criteria. Granger (1969) was one of the first to perform such an analysis. He introduced a statistical hypothesis test within the context of economics; see also his later comments (Granger 1980). It was within the Granger context that Baccalá and Sameshima (2001) introduced their novel core concept of ‘partial directed coherence’ (PDC) ensuing from their multivariate time-series analysis based on a decomposition of multivariate partial coherences. Hence, the name PDC.

This makes sense because, as Granger (1980) also pointed out himself on a later occasion, instead of talking about causality, i.e., X causes Y, the Granger causality actually tests whether X *forecasts* Y. Causality—what is that precisely?—implies prediction but probability is always dangling in the background. Neuroscience sends greetings to economics. The core concept as introduced by Baccalá and Sameshima (2001) and reconsidered by Baccalá and Sameshima (2021) is now ‘partial directed coherence’ and, for time series of spikes, the scale is neuronal.

Basal ganglia and the GPR model GPR stands for three authors, Gurney, Prescott, and Redgrave who published an anatomy-based model of action selection in what Humphries and Gurney (2021) aptly called “the dark basement of the brain,” the basal ganglia [for a clear sketch of the anatomy, see Fig. 1 of Humphries and Gurney (2021)], where an essential part of the motor program is “written.” The GPR paper does what a modern theoretical-neuroscience paper should do: Specifying the essential part of the anatomy so as to make the mathematical model, and doing the job.

Orientation selection in primary visual cortex V1 as a self-organizing process Focusing on primary visual cortex V1 of primates, von der Malsburg (1973) asked how its orientation map might come about. He devised a concrete model with 338 neurons that are described by a rate and not by a ms-time code and getting input from a “retina” of 19 cells that encode a direction. The interaction structure was patterned after the V1 anatomy in that the range of the excitatory near-neigbor interactions was shorter than that of the inhibitory interactions engulfing them. Finally, learning was going to happen in the original Hebbian sense (Hebb 1949, p 62),

*When an axon of cell A is near enough to excite a cell B and* repeatedly *or* persistently *takes part in firing it, some growth process or metabolic change takes part in one or both cells such that A’s efficiency, as one of the cells firing B, is increased.*

Hebb continued by suggesting “synaptic knobs develop.” The synapse being at an end of an axon stemming from neuron A, it has a presynaptic part and a postsynaptic part at the other side of the synaptic cleft, sitting on neuron B. So it all fit. Therefore von der Malsburg (1973) took A’s firing rate. We note, though, that timing played no role in Hebb’s proposal, whereas it is essential in STDP (Gerstner et al. 1996; Markram et al. 1997; Zhang et al. 1998). Neither does the *de*crease of synaptic strength for those spikes that “come too late,” after the postsynaptic neuron has fired.

Given the, from the present point of view, tiny size of computers at that time the project was courageous because only through numerical simulations could even a qualitative orientation map be shown to exist. Given the relatively simple but clearly structured theoretical setup, the result was astounding in that it sufficed to generate *self*-organized orientation-map formation à la Hubel and Wiesel (1962, 1963). The main contribution of von der Malsburg (1973) can be described succinctly by the title “Towards understanding the neural code of the brain” of his recent paper (von der Malsburg 2021).

Mathematically showing self-organization on the basis of known anatomical structures and mechanistic principles was an early (1973) stimulus for many and showed that mathematization works, leading to a far deeper understanding of the phenomena one wants to explain. The point is that, once a structure such as an orientation map has been shown to arise out of the chosen setup, one can quantitatively verify the influence of varying different parameters representing different aspects of the underlying structure. Assigning numerical values to parameters, the fewer the better, in a responsible manner is a technique by itself but not an issue here.

## Outlook

The above examples show both that mathematization of nature works and how it is done. Examples only sample and never depict all of natural reality, nor how scientists analyze it. After all, science is not made by abstract names but by humans. The story of Abarbanel (2021) highlights how personal interaction between the different actors in a specific domain are essential in moving the carriage of science forward. Furthermore, an intensive interaction between theory and experiment is essential to increasing our insight. Just remember Galileo’s famous, maybe even fabulous, experiment of dropping two masses of different weight from the Tower of Pisa. They arrived simultaneously at the ground and in this way refuted all previous theories.

The present paper has tried to indicate what scientific insight in the sense of mathematization of nature means: uncovering core concepts and the scale on which they act. The rest has to be filled out by the actors of science.
